# The Affective Impact of Financial Skewness on Neural Activity and Choice

**DOI:** 10.1371/journal.pone.0016838

**Published:** 2011-02-15

**Authors:** Charlene C. Wu, Peter Bossaerts, Brian Knutson

**Affiliations:** 1 Psychology and Neuroscience, Stanford University, Stanford, California, United States of America; 2 Computation and Neural Systems, California Institute of Technology, Pasadena, California, United States of America; Tel Aviv University, Israel

## Abstract

Few finance theories consider the influence of “skewness” (or large and asymmetric but unlikely outcomes) on financial choice. We investigated the impact of skewed gambles on subjects' neural activity, self-reported affective responses, and subsequent preferences using functional magnetic resonance imaging (FMRI). Neurally, skewed gambles elicited more anterior insula activation than symmetric gambles equated for expected value and variance, and positively skewed gambles also specifically elicited more nucleus accumbens (NAcc) activation than negatively skewed gambles. Affectively, positively skewed gambles elicited more positive arousal and negatively skewed gambles elicited more negative arousal than symmetric gambles equated for expected value and variance. Subjects also preferred positively skewed gambles more, but negatively skewed gambles less than symmetric gambles of equal expected value. Individual differences in both NAcc activity and positive arousal predicted preferences for positively skewed gambles. These findings support an anticipatory affect account in which statistical properties of gambles—including skewness—can influence neural activity, affective responses, and ultimately, choice.

## Introduction

Winning the lottery and contracting a life-threatening illness are both life-changing but unlikely outcomes. Most people will never experience either event, yet the profits of casinos and insurance companies indicate that individuals are willing to pay a high premium for the potential to win big or to cheat death. Although traditional economic theories have little to say about how skewed (i.e., large and asymmetric but unlikely) outcomes motivate choice, skewed outcomes may influence not only individual fortunes but also movements of the market [1].

An improved understanding of individuals' responses to skewness can inform financial theory as well as practice. Traditional economic models assume that people seek to maximize value. For instance, Blaise Pascal initially proposed that the *expected value* of an outcome could be calculated by multiplying its magnitude with its probability. Thus, expected value can be statistically approximated with the mean (the first statistical moment) of repeated gamble outcomes. Current financial models also imply that while individuals are attracted to expected value, they are instead repelled by risk. These *mean-variance* models thus approximate risk as the mathematical variance (the second statistical moment) of repeated gamble outcomes [2]. Behavioral research, however, suggests that mean-variance models cannot fully account for individuals' financial choices [3]. As a result, researchers have suggested that some choice anomalies (e.g., the lack of diversity in investors' portfolios) might result from preferences for skewness (the third statistical moment) [4]. Despite behavioral evidence that skewness can influence preferences [5], [6] either by enhancing [7] or interacting with risk [8], [9], only a few models of financial choice consider skewness. Cumulative prospect theory [10] and rank-dependent utility models [11] have attempted to account for skewness by overweighting unlikely extreme positive or negative events, but do so by sacrificing the ability to explain tolerance for variance [12].

From a psychological standpoint, statistical properties of gambles may influence choice by altering affective states [13], [14]. According to an *anticipatory affect* model, statistics can influence how individuals feel about a financial option, which can then increase or decrease their willingness to choose that option [15]. Specifically, cues signaling uncertain future gains elicit positive arousal and correlated neural activity in the nucleus accumbens (NAcc) that promotes approach. Conversely, cues signaling uncertain future losses elicit negative arousal and correlated neural activity in the anterior insula that promotes avoidance. The anticipatory affect model predicts that skewed gambles might elicit distinct patterns of self-reported affect and neural activity relative to symmetric gambles, such that positively skewed gambles (i.e., high magnitude, low probability gains) elicit greater positive arousal and NAcc activation, but that negatively skewed gambles (i.e., high magnitude, low probability losses) elicit greater negative arousal and anterior insula activation. These changes in affect and correlated neural activity may lead individuals to prefer positively skewed gambles to negatively skewed gambles.

**Figure 1:**
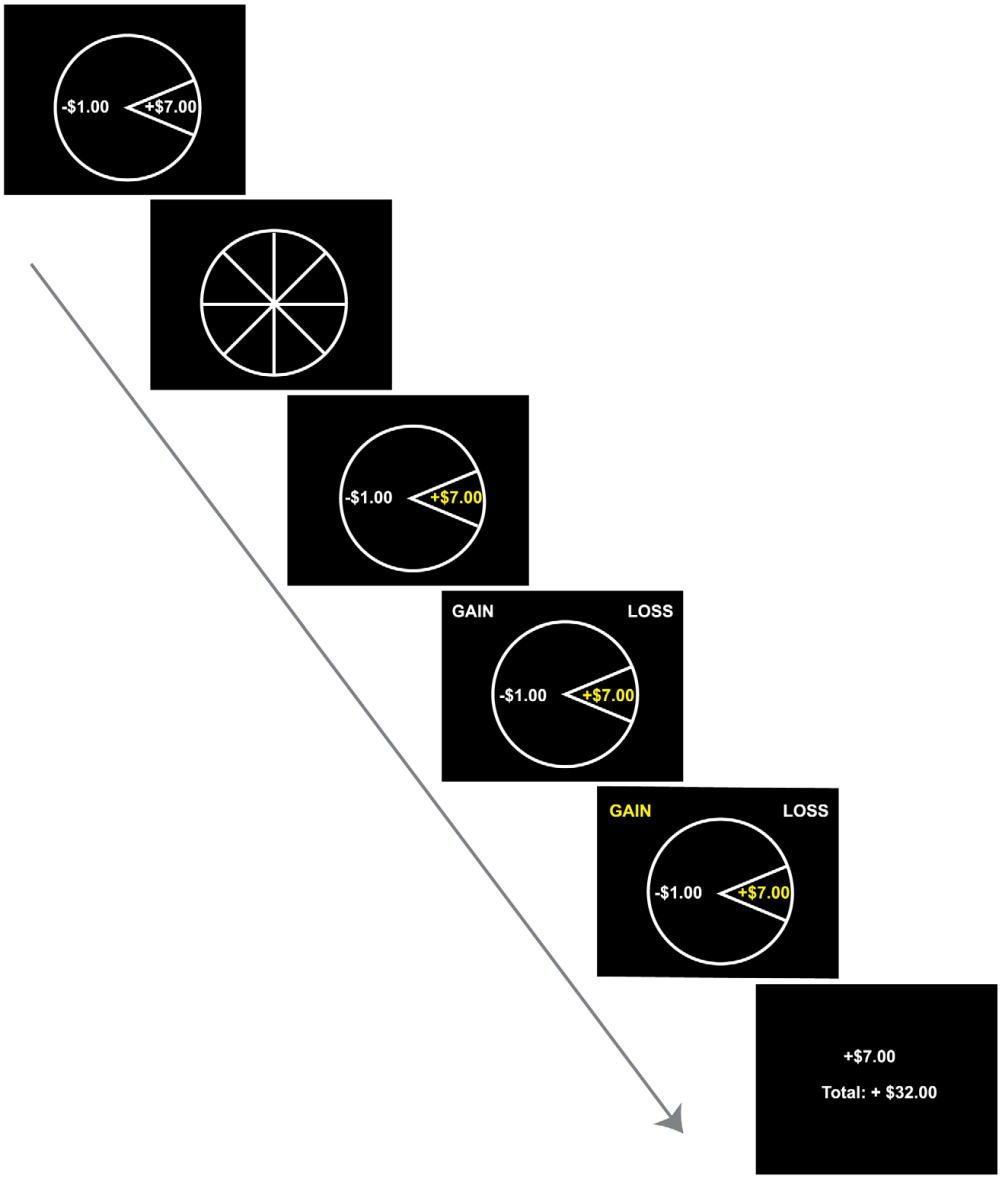
Gambling task. On each trial, subjects viewed a gamble cue, waited as the wheel spun, observed and reported the outcome (selecting “gain” or “loss” presented randomly on either side of the screen), and saw trial and cumulative earnings. Trials were separated by a centrally presented fixation cross (variable inter-trial interval; 2–6 s).

Accumulating evidence from neuroimaging studies indicates that expected value elicits NAcc activity [16], [17], whereas expected risk elicits anterior insula activity [18]–[23]. However, no studies have controlled expected value and variance to focus on the impact of skewness on financial choice. In the present study, nineteen subjects participated in a gambling game while being scanned with functional magnetic resonance imaging (FMRI), and subsequently indicated their affective reactions to and preferences for each gamble. During scanning, subjects played a series of repeated gambles that counted for actual money (Figure 1). “Low-Variance” gambles yielded an equal probability (50%) of winning or losing a small amount (±$1.00), while “High-Variance” gambles yielded an equal probability (50%) of winning or losing a moderate amount (±$2.75). “Positive-Skew” gambles yielded a low probability (12.5%) of winning a large amount (+$7.00) coupled with a high probability (87.5%) of losing a small amount (−$1.00). “Negative-Skew” gambles conversely yielded a low probability (12.5%) of losing a large amount (−$7.00) coupled with a high probability (87.5%) of winning a small amount (+$1.00). Importantly, expected value was equated across all gambles and set to $0.00 to avoid framing effects [24], while variance was equated across High-Variance, Positive-Skew, and Negative-Skew gambles, and skewness was manipulated in opposite directions for Positive- versus Negative-Skew gambles. Post-scan ratings of valence, arousal, and perceived risk were collected for each gamble type. Gamble outcome distributions ensured that all subjects received equal exposure to all gambles and experienced identical outcomes at the indicated probabilities before reporting their affective responses and preferences at the end of the experiment. To assess preference, subjects ranked their preference for the gambles and chose to play their favorite gamble again. Thus, this research represents an initial attempt to examine the influence of financial skewness on anticipatory affect, neural activity, and subsequent choice.

## Results

### Behavior

**Figure 2:**
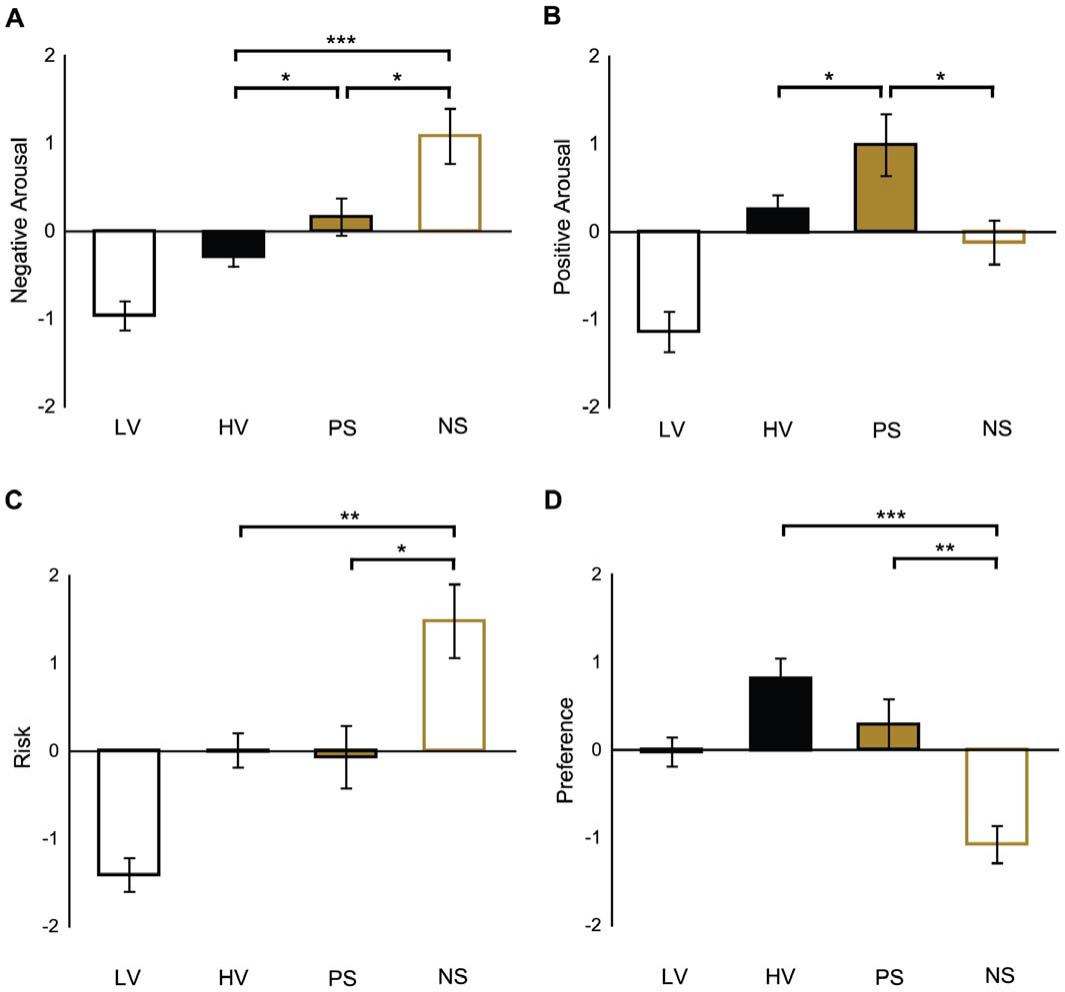
Behavioral data. Low-Variance (LV), High-Variance (HV), Positive-Skew (PS), Negative-Skew (NS) gambles. * *p*<0.05; ** *p*<0.01; *** *p*<0.001 differences between higher variance gambles. Lines represent standard errors of the mean. **A.** Negative arousal. **B.** Positive arousal. **C.** Risk. **D.** Preference.

Different gambles elicited different levels of self-reported positive arousal, negative arousal, and perceived risk (Figure 2). With respect to anticipatory affect, positive arousal varied, *F*(1, 3) = 10.78, *p*<0.001, such that all higher variance (High-Variance, Positive-Skew, Negative-Skew) gambles elicited more positive arousal than Low-Variance gambles (*p*<0.001), and Positive-Skew gambles elicited more positive arousal than High-Variance (*p*<0.05) and Negative-Skew gambles (*p*<0.05). Negative arousal also varied, *F*(1, 3) = 14.97, *p*<0.001, such that all higher variance gambles (High-Variance, Positive-Skew, Negative Skew) elicited more negative arousal than Low-Variance gambles (*p*<0.001), and Negative-Skew gambles elicited more negative arousal than High-Variance (*p*<0.001) and Positive-Skew gambles (*p*<0.05). As with negative arousal, subjects' perceived risk varied, *F* (1, 3) = 12.07, *p*<0.001, such that all higher variance gambles (High-Variance, Positive-Skew, Negative Skew) were considered riskier than Low-Variance gambles (*p*<0.001), and Negative-Skew gambles were considered riskier than both Positive-Skew (*p*<0.05) and High-Variance gambles (*p*<0.01).

Different gambles also elicited different preference rankings, *F*(1, 3) = 11.17, *p*<0.001, such that subjects preferred High-Variance to Low-Variance gambles (*p*<0.01), and preferred all other gambles to Negative-Skew gambles (*p*<0.01). Nonparametric analyses of rankings yielded similar results. Comparison of High-Variance versus Low-Variance gamble rankings using a Wilcoxon Signed-Ranks test confirmed that subjects preferred High-Variance to Low-Variance gambles (*Z* = 2.66, *p*<0.01). Contrasts of Positive-Skew and Negative-Skew gamble preferences revealed that subjects preferred Positive-Skew to Negative-Skew gambles (*Z* = 2.59, *p*<0.01) and High-Variance to Negative-Skew gambles (*Z* = 3.68, *p*<0.001). Subjects were indifferent between the two highest-ranked gambles (i.e., High-Variance and Positive-Skew; *Z* = 1.20, *n.s.*).

### Brain Activation

**Figure 3:**
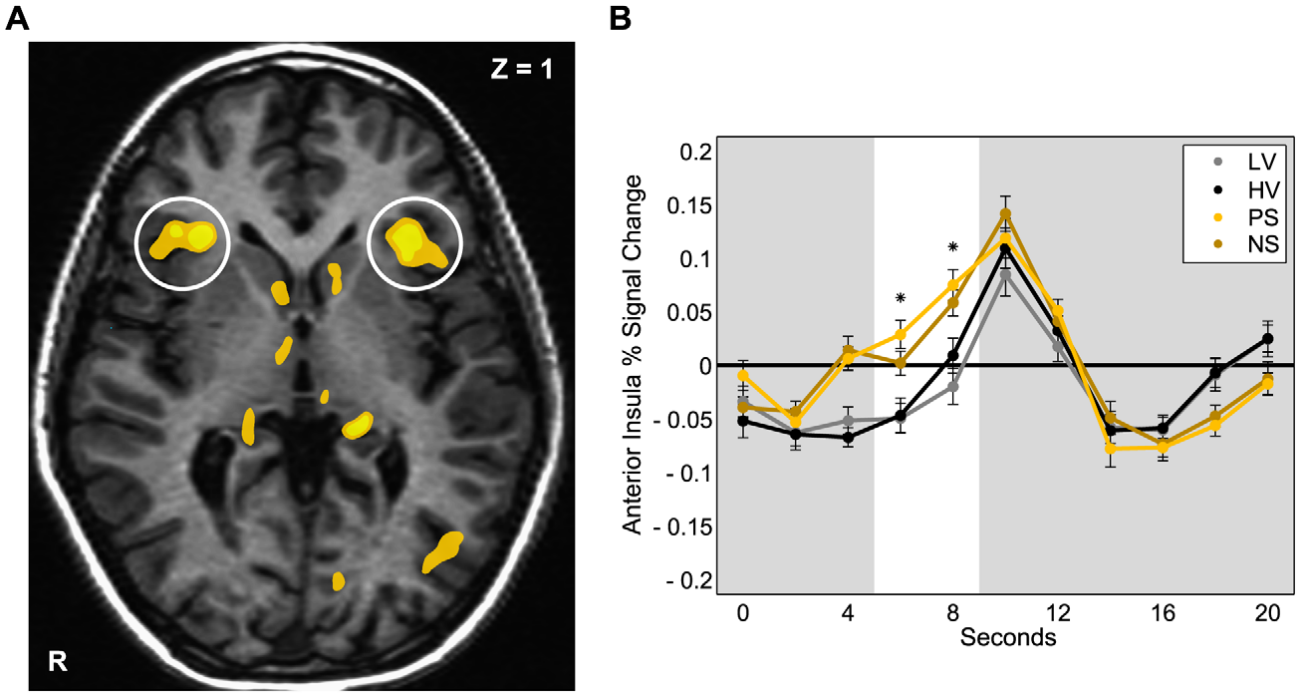
Skewness increases Anterior Insula activation. **A.** Skewed versus Symmetric; *p*<0.001 uncorrected, *p*<0.05 whole-brain corrected. (Table S1). **B.** Time courses extracted from anterior insula for gambles (*Positive- and Negative-Skew vs. Low- and High-Variance, *p*<0.05). Lines represent standard errors of the mean. The white bar highlights the anticipatory period (shifted by 6 s to account for the hemodynamic lag).

Whole-brain analyses revealed that anticipatory activation (i.e., the first 4 s of each trial) in predicted regions differed in response to variance and skewness (at a whole-brain corrected threshold of *p*<0.05) (Table S1). High variance versus low variance gambles (i.e., the contrast of High-Variance + Positive-Skew + Negative-Skew > Low-Variance) elicited increased activation in the anterior insula. Among high variance gambles, skewed versus symmetric gambles (i.e., the contrast of Positive-Skew + Negative-Skew > High-Variance) elicited even greater activation in the anterior insula (Figure 3). Among skewed gambles, positively versus negatively skewed gambles (i.e., the contrast of Positive-Skew > Negative-Skew) elicited greater bilateral NAcc activation, but at a targeted (*a priori* small volume-corrected) threshold (TC: ±12, 11, −1; *Z* = 2.30, *p*<0.05), which was further verified with timecourse analyses. Beyond group differences, individual difference analyses also indicated a link between NAcc activation and preference for Positive-Skew gambles (described below).

**Figure 4:**
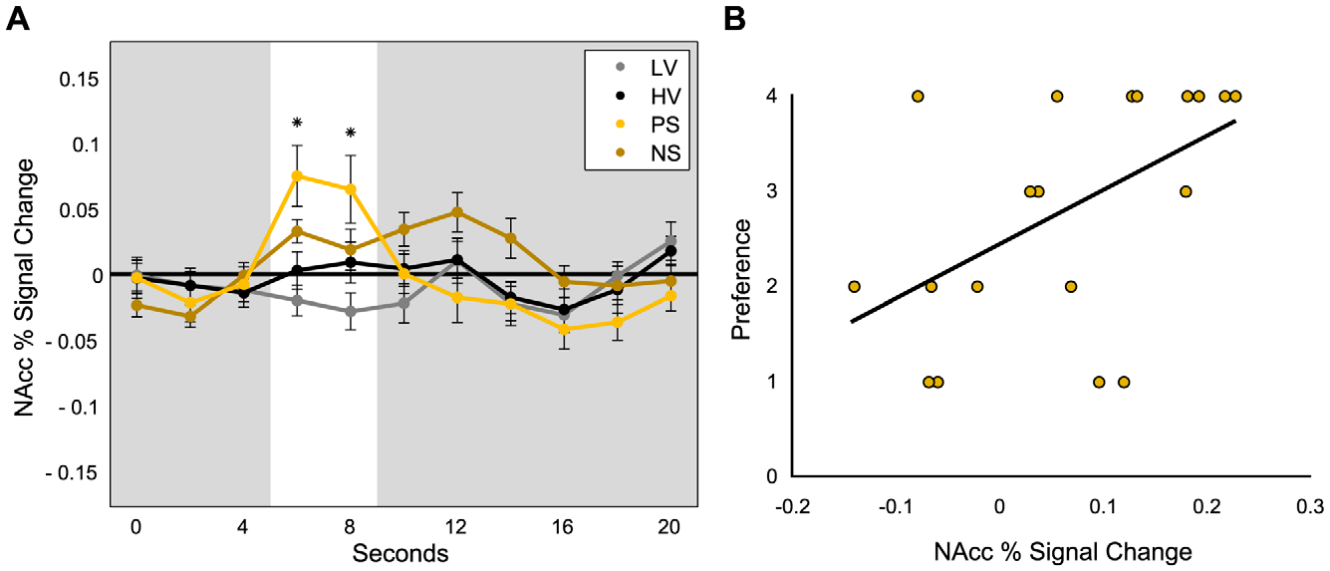
NAcc activation predicts Positive-Skew preference. **A.** Timecourses extracted from NAcc for different gambles (*Positive-Skew vs. Low-Variance, High-Variance and Negative-Skew, *p*<0.05). Lines represent standard errors of the mean. The white bar highlights the anticipatory period (shifted by 6 s to account for the hemodynamic lag). **B.** NAcc percent signal change plotted with preference for Positive-Skew gambles (*r* = 0.53, *p*<0.01).

Activation timecourses were extracted from predefined anatomical NAcc and anterior insula volumes of interest (VOI) to verify neural responses to the gambles indicated by the whole-brain analyses (Figure 3 and Figure 4). Repeated-measures ANOVAs revealed significant main effects of gamble type in anterior insula (*F* = 12.03, *df* = 3, *p*<0.001) and NAcc (*F* = 5.03, *df* = 3, *p*<0.05) activation during anticipation. Post-hoc *t*-tests (one-tailed) confirmed significant pairwise differences, such that both Positive- and Negative-Skew gambles elicited more anterior insula activation than High-Variance (*p*<0.001; *p*<0.05) and Low-Variance gambles (*p*<0.001; *p*<0.001); while Positive-Skew gambles elicited more NAcc activation than Low-Variance (*p*<0.01), High-Variance (*p*<0.05), and Negative-Skew gambles (*p*<0.05).

Consistent with previous findings indicating that NAcc activation correlates with positive arousal [25], NAcc activation selectively increased for Positive-Skew gambles. Anterior insula activation, however, increased for both Positive- and Negative-Skew gambles. Since previous research indicates that brain activation outside the context of choice can predict preference [26], [27], we examined whether individual differences in NAcc activation and anticipatory affect could account for subsequent preferences for Positive-Skew gambles. Regression models examined whether peak brain activation alone, anticipatory affect alone, and a combination of both could account for individual differences in preferences for Positive-Skew gambles. Both NAcc activation (*R* ^2^ = 0.27, *p*<0.05) and positive arousal (*R* ^2^ = 0.59, *p*<0.001) separately predicted preference. Combining both measures significantly improved the explanatory power of the model to account for individual differences in preference for Positive-Skew gambles (*R* ^2^ = 0.84, *p<*0.001). Specifically, including neural (NAcc) activation explained additional variance over and above reported positive arousal (Δ*R* ^2^ = 0.12, *p<*0.05). Anterior insula activation and reported negative arousal, however, were not significant predictors of preference for Positive-Skew gambles in this model (*R* ^2^ = 0.01, *n.s.*; *R* ^2^ = 0.13, *n.s.*).

## Discussion

The present study examined how financial skewness influences neural activity, affect, and choice. Although previous behavioral research has explored preferences for skewness [28], [29], we acquired both affective and neuroimaging measures as individuals anticipated the outcomes of skewed gambles. Beyond expected value and variance, skewness differentially elicited affect, such that positively skewed gambles increased positive arousal, but negatively skewed gambles increased negative arousal and perceived risk. Subjects also preferred positively skewed and high variance gambles to negatively skewed gambles.

These behavioral findings demonstrate that skewness influences choice in a way not accounted for by normative economic or financial models. *Expected value* and *mean-variance* approaches do not predict preference for variance and positive skewness. Further, prospect theory [30] also does not predict preference for variance, because loss aversion implies aversion to variance. The presently observed preference for variance is consistent with other studies of risky choice [31], [32], but further research is needed to determine when increasing the magnitude of monetary outcomes induces aversion to variance. Above and beyond variance, though, skewness elicited affect (indexed by positive and negative arousal) that may have promoted subsequent approach to positively skewed gambles and avoidance of negatively skewed gambles.

Previous neuroimaging studies of financial risk-taking have not systematically investigated skewness. The current findings represent a logical but novel extension of a growing neuroimaging literature examining the influence of statistical moments on choice [33], [34]. Critically, even after holding expected value and variance constant, skewed gambles elicited increased anterior insula activation. Positive skewness additionally increased NAcc activation. Further, individual differences in both positive arousal ratings and NAcc activation predicted subsequent preference for positively skewed gambles. Together, these findings imply that the impact of positive versus negative skewness may not be localized to a single brain region. Rather, skewness may act through distinct neural circuits previously implicated in anticipatory affect to influence preferences.

To systematically manipulate skewness (while holding expected value and variance constant), we used mixed gambles with an expected value of zero. Since investigators have observed differences in the attractiveness of gambles with positive and negative expected values [24], future studies that shift gambles' expected value into either gain or loss domains may determine whether behavioral and neural responses to skewness remain constant under different value frames. To collect sufficient repeated measurements for stable FMRI assessment, we examined a limited set of four gambles. Future research exploring parametric manipulations of skewness may further establish the generalizability of skewness preferences. To control for order and learning effects by ensuring that subjects had identical experiences, all gambles were initially presented and evaluated passively (i.e., in the absence of active choice). Although many previous neuroimaging studies of financial decision-making have similarly controlled expected value when manipulating risk-related variables (e.g., variance), the present findings imply that researchers might consider also controlling for skewness.

Together, these findings provide initial neural and behavioral evidence for an independent influence of skewness on financial preferences. At both group and individual levels of analysis, skewness elicited affect and neural activity that predicts future choice. Contrary to normative models that specify that higher-order moments (e.g., skewness) should influence decisions less than lower-order moments (e.g., mean, variance), these findings suggest that skewness has a disproportionately large impact on reported affective experience, neural activity, and choice. Eventually, choice may be best modeled instead by a flexible multi-attribute framework that incorporates the affective impact of gambles [35].

Because people are willing to pay a premium for positively skewed financial investments and receive a premium for shouldering negatively skewed investments [36], skewness preferences may generalize to the real world, and even influence market valuation at the aggregate level [37]. An improved understanding of how skewness preferences influence financial decision-making holds potential relevance not only to individuals who purchase lottery tickets or insurance, but also to societal reactions to improbable financial events – and by extension, to economic policy. Broadly, these findings support a dynamic and componential neuroeconomic account that may eventually connect statistical experience to affect, and ultimately, choice.

## Methods

### Subjects

Nineteen healthy native English-speaking adults (nine females; right-handed, mean age 22.0±3.28 SD) participated in the study. Subjects had no history of neurological or psychiatric disorders. Written informed consent was obtained from all subjects, under a protocol approved by the Institutional Review Board of the Stanford University School of Medicine. In addition to the 19 subjects included in the analysis, two were excluded for excessive head motion (i.e., greater than 2 mm from one whole-brain acquisition to the next) and two more were excluded for not complying with experimental instructions. In addition to a flat fee of $40.00 for two hours of participation and a $25.00 endowment, subjects' payments were determined by the outcomes during the gambling task as well the outcome of their chosen gamble at the end of the experiment (M = $66.00± SD $3.00).

### Behavioral Task and Analysis

Subjects received spoken and written instructions and completed a brief training session prior to the first experimental run in the scanner. In the gambling task, subjects played a series of repeated gambles that counted for real money. On each trial, subjects viewed the gamble (2 s), followed by a spinning wheel (2 s), and the outcome highlighted in yellow (2 s). They then pressed a button to verify the gamble outcome (2 s), and observed the cumulative total for the run (2 s). Presentation of all gambles and response prompts were counterbalanced on both right and left sides of the screen. To ensure a high response rate, failures to respond within 2 seconds resulted in an automatic loss of $0.10 in addition to the gamble outcome for that trial (i.e., a win of $1.00 would result in +$0.90 and a loss of $1.00 would result in −$1.10), consistent with methods of previous studies [21], [23], [38]. Trials were separated by brief but variable inter-trial intervals (2–6 s). The gambling task consisted of 196 total trials, during which all gambles were evaluated according to their probabilistic outcomes. Thus, this passive gambling task was designed to ensure that all subjects played the same gambles an equal number of times and experienced identical outcomes.

After completing the gambling task in the scanner, subjects rated their affective responses as they anticipated each of the gamble outcomes. Previous studies have demonstrated high concordance and reliability between online anticipatory and cued retrospective affective ratings [39]. Ratings indexed affective arousal and valence, as well as perceived risk during the anticipation phase of each gamble (first 4 seconds of a trial). All three ratings used a seven-point Likert scale, with left and right buttons indicating a continuum ranging from “not at all aroused” to “very aroused” for arousal, “very negative” to “very positive” for valence, and “not risky at all” to “very risky” for perceived risk. Ratings of gamble-elicited arousal and valence were mean-deviated within subject and plotted within a Euclidean two-dimensional space. These dimensions were then rotated by 45 degrees to derive measures of positive arousal (PA) [i.e., PA  =  arousal/sqrt(2) + valence/sqrt(2)] and negative arousal (NA) [NA  =  arousal/sqrt(2) – valence/sqrt(2)] [16], [40]. Subjective ratings of gambles were analyzed with repeated-measures analysis of variance (ANOVA), after which significant main effects were submitted to post-hoc pairwise *t*-tests to verify significant differences between gamble types, using SPSS 16.0.

### fMRI Acquisition and Analysis

Images were acquired with a 1.5T General Electric MRI scanner (General Electric, Milwaukee, Wisconsin, USA) and a standard quadrature head coil. Twenty-four contiguous axial 4-mm-thick slices (in-plane resolution 3.75×3.75 mm) extended axially from the mid-pons to the top of the skull. Functional scans were acquired with a T2*-sensitive spiral in/out pulse sequence (repetition time = 2 s, echo time = 40 ms, flip = 90 degrees) [41]. High resolution structural scans for localization and coregistration of functional data were acquired with a T1-weighted spoiled grass sequences (repetition time = 100 ms, echo time = 7 ms, flip = 90 degrees).

Analyses of neural data utilized AFNI software (National Institute of Health, Bethesda, Maryland, USA) [42]. For preprocessing, data were sinc-interpolated to correct for nonsimultaneous slice acquisition, corrected for three-dimensional motion, high-pass filtered to remove slow trends (>0.01 Hz), and normalized to percent signal change relative to the voxel mean across the task. Visual inspection of motion correction estimates confirmed that no subject's head moved more than 2 mm in any dimension from one volume acquisition to the next.

Analyses proceeded through three stages: whole-brain localization, volume of interest (VOI) time course verification, and individual difference regressions. Localization analyses utilized a multiple regression model that included independent (i.e., uncorrelated) regressors modeling anticipation of gamble outcomes: (i) high versus low variance, (ii) skewed versus symmetric, and (iii) positive versus negative skew. The model also included regressors of noninterest indexing residual motion (n = 6) and anticipation versus the rest of the trial (n = 1, unit-weighted). Maps of contrast coefficients for regressors of interest were coregistered with structural maps, spatially normalized by manually warping to Talaraich space, spatially smoothed to minimize effects of anatomic variability (FWHM = 4 mm), and collectively submitted to a one-sample t-test against the null hypothesis of no activation in order to test for group differences while controlling for random effects. The threshold for statistically significant foci activation in group maps was set at *p*<0.001 with a cluster of 16 contiguous 2.00 mm cubic voxels (i.e., minimum cluster criterion for *p*<0.05 whole-brain corrected threshold specified by AFNI's AlphaSim) [42].

For activation time course analyses, VOIs were specified as 8 mm diameter spheres centered on previously identified foci (NAcc, TC: ±11, 12, −1; anterior insula, TC: ±31, 23, 6) and confirmed by localization analyses. Activation time courses were spatially averaged within each VOI and then divided by the average activation over the course of the entire experiment to derive measures of percent signal change. To separately examine the influence of each gamble on activation in each region, VOI time courses were averaged into four conditions representing each type of gamble (i.e., Low-Variance, High-Variance, Negative-Skew, and Positive-Skew). VOI peak activations during the anticipatory period (lagged by 6 s to match the hemodynamic peak) were submitted to repeated-measures ANOVA. VOI peak activations from regions showing significant main effects were then submitted to post-hoc pairwise *t*-tests to verify differences between types of gambles.

For individual differences analyses, timecourse data during anticipation of Positive-Skew gambles were extracted from the NAcc and anterior insula for each individual. Regression analyses examined whether individual differences in peak neural activation during anticipation as well as cue-induced affect predicted subsequent preference for Positive-Skew gambles.

## Supporting Information

Table S1 **Regressors of interest Z-scores and Talaraich coordinates for peak activation foci.** Variance contrast compared high variance versus low variance gambles (High-Variance + Positive-Skew + Negative-Skew > Low-Variance). Skewness contrast compared skewed versus symmetric gambles of equal variance (Positive-Skew + Negative-Skew > High-Variance). Positive Skewness contrast compared positively skewed versus negatively skewed gambles (Positive-Skew > Negative-Skew). Regions surpassed threshold of Z>3.28 (p<0.001, uncorrected; p<0.05, whole-brain corrected; * p<0.05, small-volume corrected).
(DOC)
