# Supplementary material for: The Affective Impact of Financial Skewness on Neural Activity and Choice
Source: PLoS One. 2011 Feb 15;6(2):e16838. doi: 10.1371/journal.pone.0016838 (PMC3039661; doi:10.1371/journal.pone.0016838)
Supplement: Table S1 — Regressors of interest Z-scores and Talaraich coordinates for peak activation foci. Variance contrast compared high variance versus low variance gambles (High-Variance + Positive-Skew + Negative-Skew > Low-Variance). Skewness contrast compared skewed versus symmetric gambles of equal variance (Positive-Skew + Negative-Skew > High-Variance). Positive Skewness contrast compared positively skewed versus negatively skewed gambles (Positive-Skew > Negative-Skew). Regions surpassed threshold of Z>3.28 (p<0.001, uncorrected; p<0.05, whole-brain corrected; * p<0.05, small-volume corrected). (DOC) [file pone.0016838.s001.doc]

| **REGION** | **HIGH VS. LOW VARIANCE** | **SKEW VS. NON-SKEW** | **POSITIVE VS. NEGATIVE SKEW** |
| --- | --- | --- | --- |
| Right Mesial Prefrontal Cortex | -4.16 (4, 49, -16) |  |  |
| Left Mesial Prefrontal Cortex | -4.06 (-4, 46, -11) |  |  |
| Right Anterior Cingulate |  | 3.94 (10, 36, 16) |  |
| Right Insula | 4.46 (30, 24, 1) | 4.78 (30, 23, 1) |  |
| Left Insula | 4.17 (-30, 24, -4) | 4.83 (-33, 23, 1) |  |
| Right NAcc | 3.37 (11, 12, -1) | 3.90 (11, 11, -1) | 2.30 (11, 12, -1)* |
| Left NAcc | 3.81 (-11, 9, -1) | 3.30 (-11, 12, -1) | 2.93 (-11, 12, -1)* |
| Right Inferior Frontal Gyrus |  | 4.61 (38, 4, 26) |  |
| Left Inferior Frontal Gyrus |  | 4.27 (-38, 4, 26) |  |
| Right Posterior Cingulate | -3.95 (4, -56, 18) |  |  |
| Left Posterior Cingulate | -3.65 (-6, -56, 18) |  |  |
| Right Superior Parietal Lobule | 3.68 (24, -67, 43) | 3.99 (24, -63, 39) |  |
| Left Superior Parietal Lobule | 4.08 (-23, -58, 46) | 3.66 (-18, -64, 44) |  |
| Right Middle Occipital Gyrus | 3.90 (33, -79, 14) | 4.20 (30, -90, 18) |  |
| Left Middle Occipital Gyrus | 3.73 (-24, -86, 14) | 4.52 (-30, -86, 15) |  |
